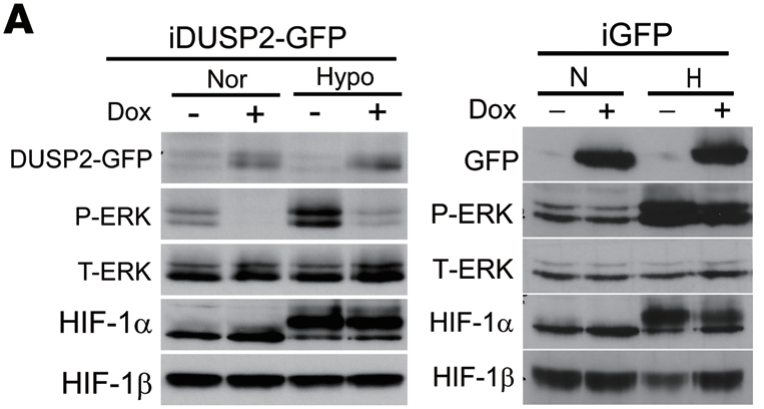# Corrigendum to Suppression of dual-specificity phosphatase–2 by hypoxia increases chemoresistance and malignancy in human cancer cells

**DOI:** 10.1172/JCI198108

**Published:** 2025-09-02

**Authors:** Shih-Chieh Lin, Chun-Wei Chien, Jenq-Chang Lee, Yi-Chun Yeh, Keng-Fu Hsu, Yen-Yu Lai, Shao-Chieh Lin, Shaw-Jenq Tsai

Original citation: *J Clin Invest*. 2011;121(5):1905–1916. https://doi.org/10.1172/JCI44362

Citation for this corrigendum: *J Clin Invest*. 2025;135(17):e198108. https://doi.org/10.1172/JCI198108

In Figure 2D of the original article, there was an error in the HIF-1β blot, which was an inadvertent duplication of the HIF-1β blot in Figure 3F. In addition, in Figure 4A, there was an error in the T-ERK blot in the iGFP panel, which was an inadvertent duplication of the T-ERK blot in the iDUSP2-GFP panel of Figure 4A. The corrected figure panels, based on the original source data, are provided below.

The authors regret the errors.

## Figures and Tables

**Figure 2D:**
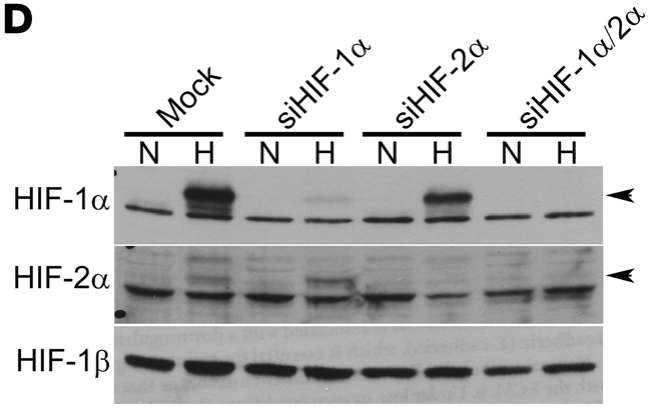


**Figure 4A:**